# Different Plant Viruses Induce Changes in Feeding Behavior of Specialist and Generalist Aphids on Common Bean That Are Likely to Enhance Virus Transmission

**DOI:** 10.3389/fpls.2019.01811

**Published:** 2020-01-31

**Authors:** Francis O. Wamonje, Ruairí Donnelly, Trisna D. Tungadi, Alex M. Murphy, Adrienne E. Pate, Christine Woodcock, John Caulfield, J. Musembi Mutuku, Toby J. A. Bruce, Christopher A. Gilligan, John A. Pickett, John P. Carr

**Affiliations:** ^1^ Department of Plant Sciences, University of Cambridge, Cambridge, United Kingdom; ^2^ Biointeractions and Crop Protection, Rothamsted Research, Harpenden, United Kingdom; ^3^ Biosciences Eastern and Central Africa, International Livestock Research Institute, Nairobi, Kenya

**Keywords:** electrical penetration graph, aphid, non-persistent transmission, potyvirus, cucumovirus, legume

## Abstract

Bean common mosaic virus (BCMV), bean common mosaic necrosis virus (BCMNV), and cucumber mosaic virus (CMV) cause serious epidemics in common bean (*Phaseolus vulgaris*), a vital food security crop in many low-to-medium income countries, particularly in Sub-Saharan Africa. Aphids transmit these viruses “non-persistently,” i.e., virions attach loosely to the insects' stylets. Viruses may manipulate aphid-host interactions to enhance transmission. We used direct observation and electrical penetration graph measurements to see if the three viruses induced similar or distinct changes in feeding behaviors of two aphid species, *Aphis fabae* and *Myzus persicae*. Both aphids vector BCMV, BCMNV, and CMV but *A. fabae* is a legume specialist (the dominant species in bean fields) while *M. persicae* is a generalist that feeds on and transmits viruses to diverse plant hosts. Aphids of both species commenced probing epidermal cells (behavior optimal for virus acquisition and inoculation) sooner on virus-infected plants than on mock-inoculated plants. Infection with CMV was especially disruptive of phloem feeding by the bean specialist aphid *A. fabae*. *A. fabae* also experienced mechanical stylet difficulty when feeding on virus-infected plants, and this was also exacerbated for *M. persicae*. Overall, feeding on virus-infected host plants by specialist and generalist aphids was affected in different ways but all three viruses induced similar effects on each aphid type. Specifically, non-specialist (*M. persicae*) aphids encountered increased stylet difficulties on plants infected with BCMV, BCMNV, or CMV, whereas specialist aphids (*A. fabae*) showed decreased phloem ingestion on infected plants. Probing and stylet pathway activity (which facilitate virus transmission) were not decreased by any of the viruses for either of the aphid species, except in the case of *A. fabae* on CMV-infected bean, where these activities were increased. Overall, these virus-induced changes in host-aphid interactions are likely to enhance non-persistent virus transmission, and data from this work will be useful in epidemiological modeling of non-persistent vectoring of viruses by aphids.

## Introduction

Aphids, predominantly the bean specialist *Aphis fabae*, but also the generalist *Myzus persicae*, are implicated in the transmission of viruses in fields of common bean (*Phaseolus vulgaris*) (Worrall et al., 2015). Among the best-studied aphid-vectored viruses of common bean are bean common mosaic virus (BCMV), bean common mosaic necrosis virus (BCMNV), and cucumber mosaic virus (CMV). BCMV and BCMNV are potyviruses with relatively narrow host ranges comprising mainly leguminous hosts (Morales, 2006; Worrall et al., 2015). In contrast, CMV is a cucumovirus with a very wide host range (Yoon et al., 2019). CMV can cause serious epidemics in common bean crops (Morales, 2006; Jacquemond, 2012), as was seen in Northeastern USA in the early 21^st^ century (Thompson et al., 2015). Introduced strains of CMV pose a potential novel threat in East Africa where common bean is a major food security crop, and where bean is already threatened by BCMV and BCMNV (Worrall et al., 2015; Mwaipopo et al., 2017; Mutuku et al., 2018; Wainaina et al., 2019).

Although all three viruses can be mechanically transmitted or transmitted through bean seed, they are most efficiently transmitted by aphids in the non-persistent manner (Bos and Maat, 1974; Davis and Hampton, 1986; Worrall et al., 2015). In non-persistent transmission, virus particles bind rapidly but loosely to receptors within an aphid's stylet (probing mouthparts) and are released during salivation (Groen et al., 2017). For non-persistently transmitted viruses such as BCMV, BCMNV, and CMV, short probes into leaf epidermal cells are considered favorable for the spread of the virus while longer probes would lead to the loss of the virus during phloem feeding (Powell, 2005; Moreno et al., 2012; Krenz et al., 2015).

Electrical penetration graph (EPG) recording is a useful technique for analyzing the feeding behavior of probing and sucking insects, many of which are important agricultural pests. The practical applications of EPG range from detection and monitoring of insect resistance to pesticides to broader biosecurity applications where the host ranges of invasive species can be determined (Garzo et al., 2016; Sandanayaka et al., 2017). Crucially, EPG has been used to decipher complex plant-pathogen-vector interactions occasioned by the ability of microbes to alter the behavior of insect vectors such as aphids, whiteflies, and psyllids to benefit their transmission (Bonani et al., 2010; Ziebell et al., 2011; Moreno-Delafuente et al., 2013; Westwood et al., 2013; Carmo-Sousa et al., 2014). For example, in cucurbits and *Arabidopsis thaliana*, CMV infection causes accumulation of plant metabolites that are distasteful to aphids (Mauck et al., 2010; Westwood et al., 2013). These distasteful compounds deter aphids from settling and encourage their dispersal, which will accelerate virus transmission to plants in the immediate vicinity (Donnelly et al., 2019). EPG measurements showed that aphids on CMV-infected Arabidopsis plants ingested less phloem sap, which would normally be these insects' major nutrition source and aphids confined on these plants grew less well (Westwood et al., 2013). When aphids were moved from CMV-infected plants to healthy plants, their growth rate recovered which was indicative that CMV used feeding deterrence and not host toxicity as the mechanism to render the plants as unsuitable hosts for aphids (Westwood et al., 2013). Epidemiologically, this is an important scenario because virus-induced changes in feeding habits can affect virus acquisition and inoculation by aphids (Mauck et al., 2012; Mauck et al., 2016; Carr et al., 2018). We investigated how three different viruses of common bean (BCMV, BCMNV, and CMV) influence the behavior of a specialist and non-specialist aphid by examining the effects of virus infection on aphid feeding behavior.

## Materials and Methods

### Viruses, Plants, and Insects

BCMV isolate PV-0915, BCMNV isolate PV-0413, and a bean-infecting isolate of CMV (PV-0473) were obtained as freeze-dried infected leaf tissue from the Deutsche Sammlung von Mikroorganismen und Zellkulturen GmbH (DSMZ) (German Collection of Microorganisms and Cell Cultures). Bulking of inoculum was done by sap inoculation of *Nicotiana benthamiana* plants before passaging the viruses to common bean. Virus-inoculated *N. benthamiana* plants were cultivated for least 3 weeks following inoculation and systemically infected leaves were harvested and stored at −80°C for use in subsequent sap inoculation of bean plants.

### Growth Conditions and Virus Inoculation of Bean Plants

Experiments were conducted with the common bean (*Phaseolus vulgaris* L.) variety Red Haricot-GLP 585 cv. “Wairimu” (SimLaw Seeds, Nairobi, Kenya), which is susceptible to infection by BCMV, BCMNV, and CMV. Bean seeds were germinated at 25°C for 5 days in a Petri dish lined with moistened filter paper and after germinating, single beans were planted in Levington M3 compost (Scotts, Chilworth, UK) mixed with sand (J. Arthur Bowers, Lincoln, UK) in a 4:1 ratio in round pots 100 mm × 90 mm (diameter × depth). Bean plants were grown in a growth room (Conviron, Manitoba, Canada) under a long photoperiod (16-h light and 8-h darkness), with a light intensity of 200 µE.m^−2^.s^−1^ (Sylvania Activa 172 Professional 36-W bulbs), at 20°C –22°C.

Once grown to the two-leaf stage (5 days post potting), plants were either inoculated with virus or mock-inoculated with water. For sap inoculation, 50-mg frozen leaf was ground in 1-ml distilled water using a pestle and mortar. Two lower leaves were dusted with Carborundum (SiC), which was used to abrade the leaf during mechanical inoculation to aid virus entry. The sap was rubbed gently onto the two leaves and then the excess sap and ground leaf debris cleaned off by spraying the leaf with distilled water. The plants were left to grow for another 10 days and by then the virus-infected plants displayed clearly observable disease symptoms (see **Supplementary Figure 1**). EPG experiments were conducted at 10 days post-inoculation.

### Verification of Plant Infection by RT-PCR

For further confirmation of infection, RT-PCR was done using plant leaf samples post-EPG experiments. Testing prior to the experiment was avoided as introduction of injury to the leaves could have triggered wound-induced changes to the plant physiology that could affect aphid herbivory. Briefly, approximately 50 mg of fresh symptomatic, leaf samples were obtained by using a 1-cm-diameter cork borer. RNA extraction was done using a total RNA purification kit (Norgen Biotek, Thorold, Ontario, Canada) using the manufacturer's instructions. RNA quality and quantity were measured using a Nanodrop^®^ ND1000 spectrophotometer (Thermo Fisher Scientific, Waltham, Massachusetts, USA). RNA was reverse-transcribed using GoScript™ (Promega) reverse transcription kits as per manufacturer instructions.

For BCMNV (isolate PV-0413; GenBank number HG792063), the sequences for the primers used to detect the BCMNV coat protein coding sequence (CP) were reverse primer 5′-AGA GAA TAT TCA TAC CCGC-3′ and 5′-ACA CAA GAG CTA CCA AG-3′ as forward primer. For BCMV (isolate PV-0915, GenBank Number: HG792064), the sequences for the BCMV CP gene-specific primers were forward primer 5'-TGA CAA TGG CAC TTC ACC-3' and reverse primer 5'- AACAAACATTGCCGTAGC-3'. The technique used to design these primers is described in an earlier publication (Mutuku et al., 2018). The primers for the CMV CP gene (isolate PV-0473; GenBank number; MH748553.1) were forward primer 5'-ACC ATC TCC TAG GTT TCT TCGG-3' and reverse primer 5'- GTC TCC TTT TGG AGG CCC-3'. Another CMV CP gene-specific primer set was also used: forward primer 5′-ATG GAC AAA TCT GAA TCA ACC AGT GCT-3′ and reverse primer 5′-TCA GAC TGG GAG CAC TCC AGA TGT GGG-3′ (Kwon et al., 2016). PCR conditions were 94°C for 3 min followed by 35 cycles of 94°C for 30 s, 56°C for 30 s, 72°C for 1 min, and a final 5-minute extension at 72°C. For visualization of successful PCR amplification, the amplicons were loaded into wells of a 1% (w/v) agarose gel in TAE containing 0.05 µg. ml-1 ethidium bromide stain. The gels were submerged in TAE buffer and run in an MHU–1010 gel rig (Flowgen/Scientific Laboratory supplies, Hessle, UK) at 100 V using a Power-Pac 3000 (Bio-Rad, Hemel Hempstead, UK). A 100-bp DNA ladder (Bioline, London, UK) was used to facilitate size estimation. Gels were examined under UV illumination on a gel documentation system to determine if the amplicons were of the expected product sizes (approximately 800 bp).

### Rearing of Aphids

Experiments used two aphid (*Aphididae*: *Hemiptera*) species: *Myzus persicae* Sulzer (common names: peach-potato or green peach aphid), and *Aphis fabae* Scopoli (common name: black bean aphid). Both colonies were generated by transferring a single aphid to a host plant. The insecticide-sensitive *M. persicae* clone US1L (Devonshire and Sawicki, 1979) was maintained on Chinese cabbage (*Brassica rapa* subspecies *pekinensis*) cv. Green Rocket (Tozer Seeds, Cobham, UK). The Kennedy and Booth clone of *A. fabae* (Kennedy and Booth, 1950) was maintained on broad bean (*Vicia faba* L.) cv. Sutton dwarf (King Seeds, Essex, UK). Aphid stock colonies were maintained on plants in individual pots in a growth chamber at 22°C under long day conditions and subsequently passaged to new plants every 2 weeks. Infested plants were covered with micro-perforated bread bags (Seal Packaging, Luton, UK) secured around the pots with rubber bands to contain the aphids.

### Observations of Aphid Feeding Behavior

To observe aphids' initial probing behavior, we used a previously described direct observation method (Caillaud et al., 1995). Adult *A. fabae* or *M. persicae* were starved for 30 min prior to experimentation. Single aphids were placed on the adaxial surfaces of leaves, observed under magnification, and the time taken for each aphid to first insert its stylet was measured.

More detailed observations of aphid feeding behavior were done using the EPG method (Tjallingii, 1978; Tjallingii and Esch, 1993; Tjallingii, 2006) as previously described (Ziebell et al., 2011; Westwood et al., 2013). Briefly, individual aphids were starved for 30–60 min before being tethered to approximately 4 cm lengths of 20 µm diameter gold wire (EPG systems, Wageningen, The Netherlands) using conductive silver paint (EPG systems), which was soldered to a 1-cm brass pin, connected to an amplifier with 1 GΩ resistance and 50–100X gain. Connected aphids were placed on individual plants inside a Faraday cage and signals received from the EPG monitor taken over 8-h recording periods and 15 aphids per treatment. EPG signals were analyzed using A2EPG software (Adasme-Carreno et al., 2015) and automated EPG parameter calculations used Microsoft Excel-based spreadsheets (Sarria et al., 2009).

### Statistical Analyses

Two types of statistical analyses were used. In the first, generalized linear models (GLMs) were used to model the number of occurrences of a waveform and to model the duration of waveforms. In the second, survival analysis was used to model the rate at which waveforms were entered.

We modeled EPG data that took the form of waveform durations using GLMs with Gamma-distributed response variables. We modeled EPG data that took the form of the number of occurrences of waveforms using GLMs with either Poisson- or negative binomial-distributed response variables. In all cases, the GLM included virus treatment as a fixed effect. In preliminary analyses of the count data (number of occurrences), dispersion tests were conducted for each waveform, and where the data was found to be significantly over-dispersed a negative binomial distribution GLM was used (otherwise a Poisson GLM was used). In addition, we modeled potential E2 index (PEI) (i.e., the proportion of the EPG recording time remaining to an aphid, following completion of a first E2 waveform, that is spent in subsequent E2 waveforms) using a GLM with Beta-distributed response variable. Note, that while a Beta distribution was the most appropriate for proportional data of this type, it is, however, necessary to perform the data transformation,

$$Y\;=\frac{\left(y\;\left(n-1\right)+\;0.5\right)}{n},\;$$

where *Y* is the transformed response variable, *y* is the original response variable and *n* is the sample size (Smithson and Verkuilen, 2006). The transformation is necessary because the Beta distribution is defined for the open interval (0, 1) while the PE2 data contains “zeros” and “ones”. In all of the above cases (duration, count, and proportion data), *post hoc* comparisons were made using the *multcomp* package in R (Hothorn et al., 2009), through the extraction of Dunnett contrasts with Bonferroni adjustments (i.e., to test for significant differences between counts, durations, or proportions, on virus-treated plants compared with mock-inoculated plants).

Data of the “time-to” form, i.e., time-to-first probe, and time to phloem EPG phases E1 and E2, were analyzed by survival analysis: a method of analyzing the occurrence of an event during an observation period. Survival curves were estimated by the Kaplan-Meier method (survival package in R, surv, and survfit functions: Therneau, 2015). Virus treatment curves were compared with mock treatment curves using the Peto-Peto test (survminer package in R, surv_pvalue function: Kassambara et al., 2017) to evaluate if virus infection affected the time taken by aphids to begin probing on the leaf epidermis and initiate phloem ingestion respectively. All analyses were conducted in R (version 3.5.0) (R Core team, 2014).

## Results

### Aphid Probing Behavior is Altered on Virus-Infected Bean Plants

**Figure 1:**
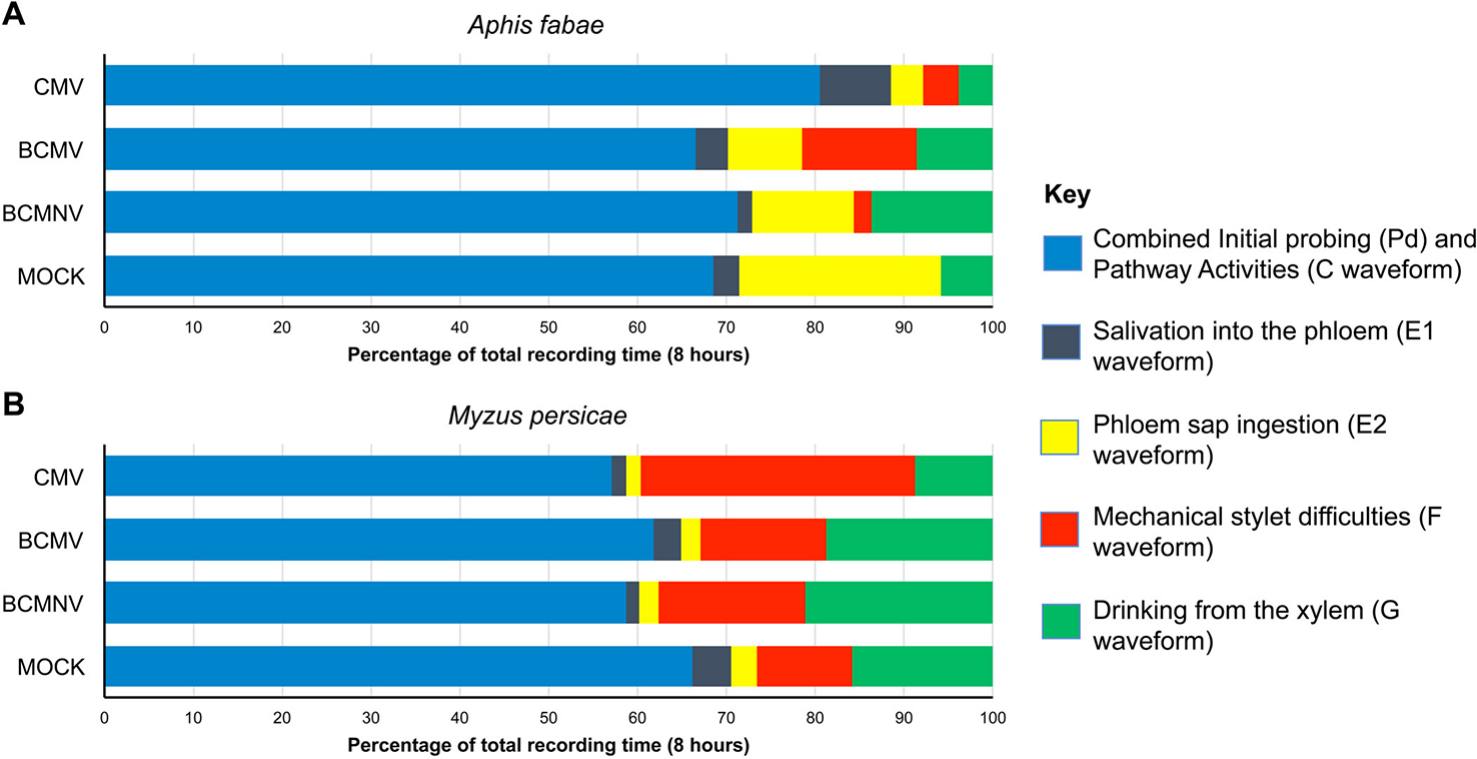
Electrical penetration graph (EPG) monitoring showed that feeding behavior of specialist and non-specialist aphids was modified on virus-infected bean plants. For both *Aphis fabae* **(A)** and *Myzus persicae* **(B)**, the combined time engaged in epidermal cell probing and pathway activity accounted for most of the activity recorded over 8 h and this was not markedly altered by the virus infection status of plants, except in the case of *A. fabae* placed on plants infected with cucumber mosaic virus (CMV), where these activities were increased **(A)**. Phloem ingestion accounted for a substantial proportion of *A. fabae* activity on mock-inoculated plants **(A)** but this was not the case for *M. persicae* where mechanical stylet difficulties and drinking from the xylem occurred, indicating that common bean is a poor host for *M. persicae* **(B)**, which is a generalist aphid, rather than a legume specialist like *A. fabae*. Phloem ingestion by *M. persicae* was not markedly affected on plants infected with bean common mosaic virus (BCMV), bean common mosaic necrosis virus (BCMNV), or CMV but mechanical stylet difficulties increased, especially on CMV-infected plants **(B)** and for *A. fabae* phloem ingestion declined on virus-infected plants **(A)**. Data was collated from EPG recordings of 240 aphids comprising 15 aphids per treatment for each aphid species, i.e., n (*M. persicae*) = 120, and n (*A. fabae*) = 120.

**Figure 2:**
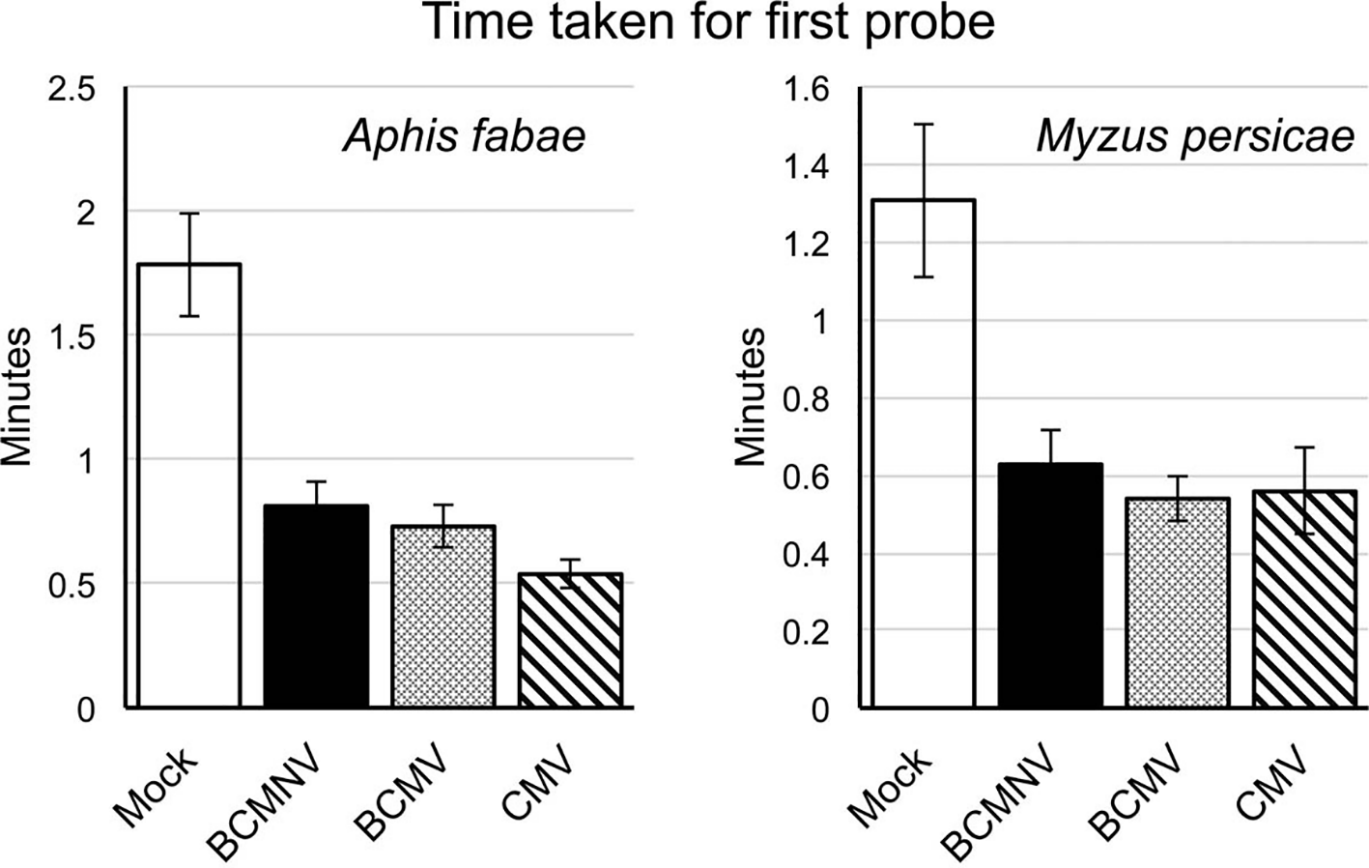
Aphids placed on virus-infected plants initiate probing behavior sooner than on mock-inoculated common bean plants. Direct observations showed that both the legume specialist *Aphis fabae* and generalist *Myzus persicae* began probing on virus-infected plants sooner than on mock-inoculated plants. Bar charts show the mean times from placement to first probe for 40 aphids per treatment group to begin probing. All experiments were done using single aphids placed on separate plants (virus-infected or mock-inoculated plants at 10 days post-inoculation/mock inoculation). Error bars represent the standard error of the mean. The decreased times-to-probe for aphids on virus-infected plants compared to mock-inoculated plants were statistically significant in all cases [survival analysis, Kaplan-Meier: *p* < 0.0001 (*A. fabae*) and *p* = 0.00012 (*M. persicae*)]. Pairwise comparisons between aphid behavior on mock-inoculated and infected plants showed significant differences [Peto-Peto: BCMNV vs. Mock *p* = 0.00018, BCMNV vs. Mock *p* = 4.88.10^−5^, CMV vs. Mock *p* = 2.45.10^−7^ (*A. fabae*)] and [Peto-Peto: BCMNV vs. Mock *p* = 0.005, BCMNV vs. Mock *p* = 0.00093, CMV vs. Mock *p* = 0.00026 (*M. persicae*)]. Mock, mock-inoculated plants; CMV, cucumber mosaic virus-infected plants; BCMV, bean common mosaic virus-infected plants, and BCMNV, bean common mosaic necrosis virus-infected plants.

Aphid activities associated with probing, salivation, feeding from the sieve elements, mechanical stylet difficulties, and drinking from the xylem were recorded by EPG for 8 h. The different waveforms observed and the associated aphid behaviors are shown in **Table 1** and **Supplementary Figure 2**. For aphids of both species, the combined probing (Pd) and stylet pathway (C) activities (indicated in blue) accounted for over 50% of total recorded activity in all treatments (**Figure 1**). For *A. fabae*, the highest proportion of time spent in stylet pathway activity (waveform C) was on CMV-infected plants (80.5%), while for *M. persicae*, it was highest on mock-inoculated plants (66.3%). Over the total EPG recording period, *A. fabae* spent proportionally more time in phloem related activities (30.6%) than *M. persicae* (7.2%) on mock-inoculated plants (**Figure 1**). The calculated statistical outputs from the GLM analyses of the occurrence and duration of the different activities inferred from the recorded waveforms for *A. fabae* and *M. persicae* is provided in the additional material (**Supporting Information: Excel Files 1 and 2**, respectively). In addition to EPG studies, visual observations of aphid probing were carried out. These showed that individuals of both aphid species started probing earlier on virus-infected plants than on uninfected plants. Pairwise *post hoc* comparisons showed significant differences in time taken by aphids to begin probing on virus-infected plants when compared to mock-inoculated plants but not among the virus treatments (**Figure 2**).

**Table 1:** Summary of electrical penetration graph (EPG) waveforms used in data analysis.

EPG waveform	Correlation		Comments
	Plant Tissues	Aphid activity/Behavior	
**NP**	Stylet not inserted	Non-penetration	Includes aphid walking on the leaf
**C**	All tissues	Activity during stylet pathway	Associated with aphid stylet penetration of the leaf cuticle, sheath salivation and other pathway activities. Is sometimes combined with the Pd waveform in analyses
**Pd**	All living cells	Stylet puncture of cell membrane	Associated with non-persistent virus acquisition and inoculation
**E1**	Phloem	Saliva secretion	Associated with virus inoculation for persistently-transmitted viruses (but not for *non-persistently* transmitted viruses)
**E2**	Phloem	Sap ingestion	Associated with plant acceptability
**F**	All tissues	Mechanical stylet activity	Penetration difficulty
**G**	Xylem	Active sap ingestion	Associated with drinking water due to dehydration

Based on Tjallingii (1978) and Tjallingii and Esch (1993).

From the EPG observations, the periods of time during which aphids were not probing were significantly shorter for *A. fabae* on BCMV-infected plants (*p* = 0.00057) when compared to mock-inoculated plants (**Table 2**). This was attributed to a significant reduction in total duration spent in the pathway phase (C) (*p* < 0.001), as there were no significant differences between the different treatments in the number of potential drops (Pd). *M. persicae* probed less frequently on virus-infected plants with statistically significant reductions for those individuals placed on plants infected with BCMNV (*p =* 0.022) or BCMV (*p =* 0.024) when compared to those monitored on mock-inoculated plants.

**Table 2:** Probing and pathway behavior (mean ± SEM) of *M. persicae* and *A. fabae* on leaves of virus-infected and mock-inoculated (Mock) bean plants over an 8-h EPG recording.

Aphid	EPG Parameters (waveform^#^)		Unit	Mock^§^	BCMNV	BCMV	CMV
***Aphis fabae***	**General probing behavior**	Duration spent not probing (Np)	Min	101 ± 18.8	125 ± 23.9	228 ± 15.9* [*p* = 0.00057]	112 ± 15.8
		Number of probes	Number	17 ± 2.3	18 ± 2.4	22 ± 3	22 ± 2.7
		Number of short probes (C < 3 min)	Number	7.4 ± 1.4	8.6 ± 2	12 ± 2.5	10 ± 1.9
	**Pathway phase** **(C plus Pd)**	Total duration of pathway (C)	Min	249 ± 20.4	242 ± 19.8	118 ± 15.1* [*p* = 1.52.10^-8^]	296 ± 16
		Number of Pds	Number	163 ± 16	158 ± 17	165 ± 15	221 ± 16
***Myzus persicae***	**General probing behavior**	Duration spent not probing (Np)	Min	127 ± 13.5	115.7 ± 18.7	106.7 ± 15.8	110 ± 14
		Number of probes	Number	50 ± 5.8	32.1 ± 4.5* [*p* = 0.022]	32.3 ± 3.8* [*p* = 0.024]	41 ± 5
		Number of short probes (C < 3 min)	Number	32.3 ± 5	18.9 ± 3.8	20.4 ± 3.3	25.5 ± 4.3
	**Pathway phase** **(C plus Pd)**	Total duration of pathway	Min	231 ± 11.8	209 ± 11.8	226 ± 14.1	212 ± 19.2
		Number of Pds	Number	186 ± 10.8	187 ± 15.4	222 ± 16.3	185 ± 16.7

Asterisk (*) indicates that the Bonferroni corrected *p* value, associated with behavioral comparisons on virus-treated vs mock inoculated plants, was significant to the 0.05 level (p-values were obtained using Dunnett post hoc tests from GLM models, see statistical analyses, main text).

**^#^**For full descriptions of EPG waveforms see **Table 1** and **Supplementary Figure 2**.

^§^Mock, mock-inoculated *vs.* plants infected with bean common mosaic necrosis virus (BCMNV), bean common mosaic virus (BCMV), or cucumber mosaic virus (CMV).

### *A. fabae* Phloem Feeding is Inhibited on Virus-Infected Plants While *M. persicae* Appears to Find Bean Unfavorable for Phloem Feeding

**Figure 3:**
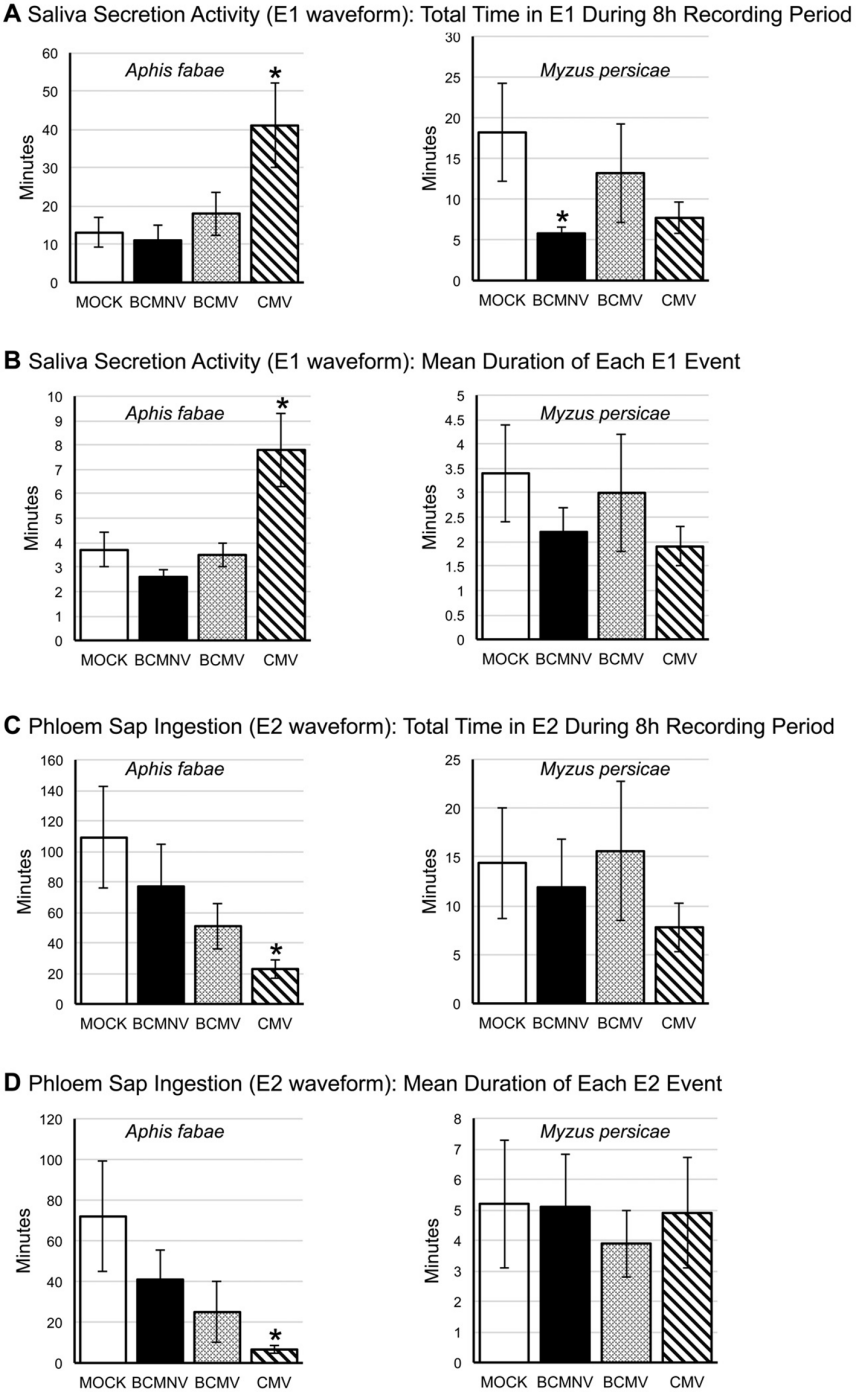
Analysis of differences in aphid salivation into the phloem and phloem sap ingestion for *Aphis fabae* and *Myzus persicae* on mock-inoculated and virus-infected plants. EPG data was analyzed for changes in salivation into the phloem (E1 waveform: **A, B**) and subsequent phloem sap ingestion (E2 waveform: **C, D**) by the legume specialist *A. fabae* (left panels) and the generalist *M. persicae* (right panels) on common bean plants that had been mock-inoculated or infected with bean common mosaic virus (BCMV), bean common mosaic necrosis virus (BCMNV), or cucumber mosaic virus (CMV). *A. fabae* placed on CMV-infected plants spent significantly longer (*p =* 0.0161) salivating into the phloem than on plants that were mock-inoculated **(A)**, and this was consistent with a corresponding significant difference for the mean duration of incidents of sap ingestion (*p =* 0.0236) **(B)**. The total time *A. fabae* spent phloem feeding was significantly reduced on CMV-infected plants (*p =* 0.00059) **(C)** as was the mean duration of phloem ingestion bouts (*p =* 8.0.10^−5^) **(D)**. *A. fabae* on plants infected with BCMV showed a similar trend of decreased phloem activity, although this was not statistically significant **(A–D)**. On mock-inoculated plants *M. persicae* and *A. fabae* spent similar periods of time salivating into the phloem **(A, B)**. However, on mock-inoculated plants, the phloem ingestion bouts of *M. persicae* averaged 5 min compared to 70 min for *A. fabae* **(D)**, which was reflected in the overall times spent feeding from the phloem by the two species **(C)**. The only statistically significant effect seen for *M. persicae* was a decrease in overall time spent in salivation on BCMNV-infected plants (*p =* 0.0427) **(A)**. Asterisks denote values significantly different from mock treatments (Dunnett *p* < 0.05). The error bars represent the standard error of the mean.

More detailed examination of EPG data for *A. fabae* pre-ingestion salivation activity into the phloem (E1 waveform) and subsequent phloem ingestion (E2 waveform) showed that on CMV-infected plants there were significant changes to feeding behavior when compared to aphid activity on mock-inoculated plants. Though the number of E1 events were not significantly increased for *A. fabae* aphids foraging on CMV-infected plants, there were significant increases in both the total duration (*p =* 0.0161) and mean length (*p* = 0.00236) of E1 activities (**Figure 3**). This is indicative of difficulty feeding from the phloem. Also, both the total duration and mean length of E2 was significantly reduced for *A. fabae* on CMV-infected plants (**Figure 3**).

**Figure 4:**
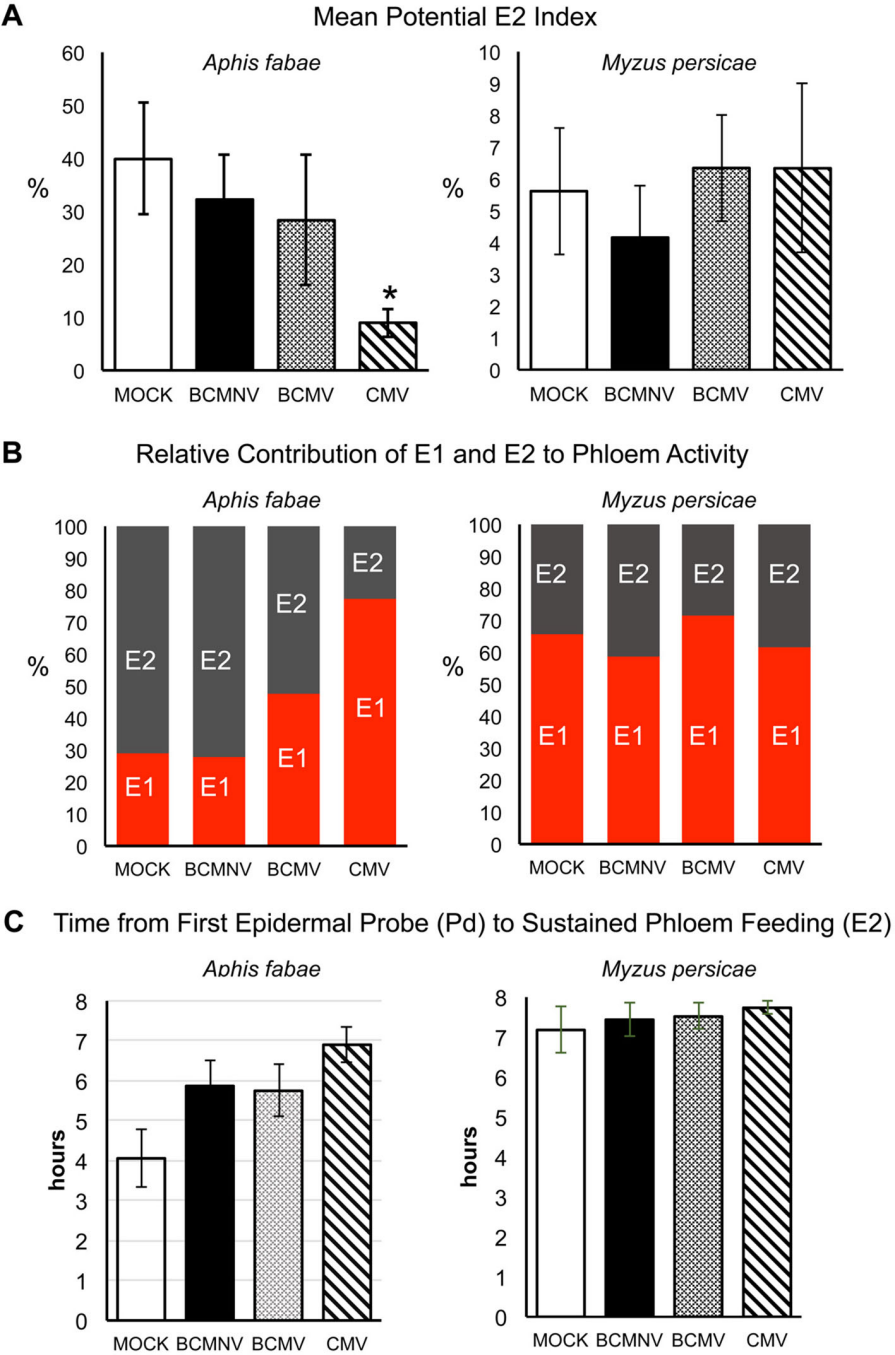
On common bean *Myzus persicae* is less likely than *Aphis fabae* to ingest phloem sap and its phloem feeding activity is less affected by plant infection status. **(A)** Using EPG data the likelihood (as percentage odds) of an aphid transitioning to sustained phloem sap ingestion (the potential E2 index, PEI) was determined by beta GLM with the multcomp package in R (Hothorn et al., 2009) for *A. fabae* and *M. persicae* placed on mock-inoculated plants or plants infected with bean common mosaic virus (BCMV), bean common mosaic necrosis virus (BCMNV), or cucumber mosaic virus (CMV). Sustained phloem feeding in this case is indicated by periods of E2 waveform activity of >10 min. **(A)** As shown by the PEI, *A. fabae* was markedly more likely to ingest phloem sap from mock-inoculated plants than *M. persicae* (which appears to find common bean an unsuitable host) but the likelihood of phloem feeding was diminished for *A. fabae* on virus-infected plants and was significantly decreased on CMV-infected plants (*p =* 0.040). Virus infection status had no effect on the already low likelihood of phloem feeding by *M. persicae*. **(B)** On plants infected with BCMV and CMV but not with BCMNV, the proportion of time spent by *A. fabae* in salivation into the phloem (waveform E1) relative to sap ingestion (waveform E2) increased markedly. The proportion of time *M. persicae* spent salivating into or feeding from the phloem appeared unaffected by plant infection status. **(C)** EPG recordings of *A. fabae* on mock-inoculated plants showed sustained phloem feeding began by 4-h post-placement while *A. fabae* placed on plants infected with BCMV, BCMNV, or CMV took on average 5.8, 5.7, and 6.9 h, respectively. In all panels error bars represent standard error of the mean. The asterisk denotes a significant difference in feeding behavior between insects on virus-treated and mock-inoculated plants (Dunnett test, p < 0.05: see **Supplementary Table 3** for analyses).

An analysis of the likelihood that sustained continued phloem feeding would occur (PEI), showed that *A. fabae* aphids were less likely to feed from the phloem of virus-infected plants. The likelihood of recurrent phloem feeding on uninfected plants was highest for aphids on uninfected plants (39.85%) as compared to BCMNV (32.22%), BCMV (28.31%), and CMV (8.97%) (**Figure 4**). Pairwise comparisons were significantly lower for aphids on CMV-infected plants (*p* = 0.040) (**Figure 4**, **Supporting Information Excel File 3**). Notably, the E1 events exhibited by *A. fabae* contributed to more than half of all phloem phase activity for aphids on CMV-infected plants. *A. fabae* took longer to transition from phloem salivation to sustained phloem feeding (where E2 lasted longer than 10 min). On mock-inoculated plants, *A. fabae* averaged 3.11 h before their first sustained phloem feeding event, which increased to 4.11, 4.67, and 4.92 h on BCMNV, BCMV, and CMV-infected plants, respectively (**Figure 4**).

By comparison, there was no discernible trend in the PEI from recordings of *M. persicae* on virus-infected and mock-inoculated plants (**Figure 4**). The mean PEI for *M.* persicae on mock-inoculated plants was 5.6%, which is lower than the lowest PEI for *A. fabae*, which was 8.8% on CMV-infected plants. Thus, the generalist aphid *M. persicae* is probably less likely to settle and feed on common bean plants than the bean specialist *A. fabae*, regardless of the host's virus infection status. The E1 salivation events exhibited by *M. persicae* contributed to more than half of all phloem phase activity for all four treatments (Mock: 65%, CMV: 61%, BCMNV: 58%, and BCMV: 71%), and sustained phloem feeding events were recorded towards the end of the 8-h recording period (**Figure 4**).

### *A. fabae* and *M. persicae* Displayed Differences in Phloem Feeding Behavior That Were Attributable to Host Suitability as Well as to the Presence of Virus Infection

**Figure 5:**
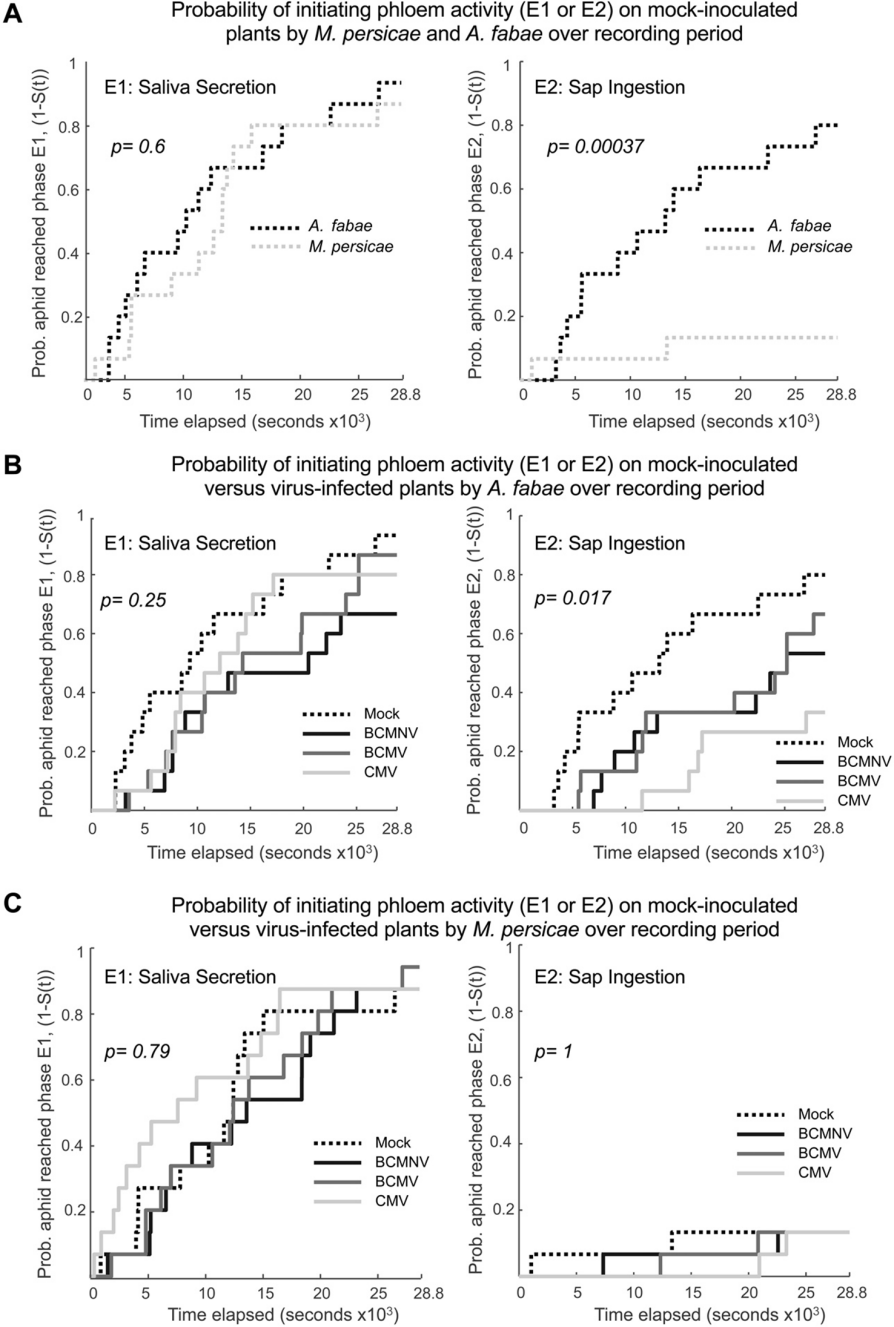
The rate at which *Aphis fabae* engage in repeated phloem sap ingestion is decreased on virus-infected plants, whereas *Myzus persicae* is reluctant to ingest phloem sap regardless of plant infection status. The EPG E1 (saliva secretion into the phloem) and E2 (ingestion of phloem sap) waveform data for both *A. fabae* and *M. persicae* were subjected to survival analysis, which is an application of an actuarial method for assessing the probability that a specific event will occur during a given period of observation time. Curves representing the probability that aphids had re-entered the phloem and were producing waveforms related to phloem feeding (i.e., survival curves, the probability that the aphid did not feed as a function of time, S(t), denoted as 1-S(t) on the Y-axes) were computed for each waveform. **(A)**. On mock-inoculated plants, the likelihood of aphids initiating saliva secretion (E1 activity, left panel) was similar for the legume specialist *A. fabae* and for the generalist *M. persicae*. However, the probability of transitioning back to phloem sap ingestion (E2 activity, right panel) was significantly lower for *M. persicae* than for *A. fabae*. When feeding on virus-infected plants (infected with either BCMV, BCMNV, or CMV), there was no significant decrease in the probability of either *A. fabae* **(B)** or *M. persicae* **(C)** initiating saliva secretion into the phloem (E1 activity, left panels). However, *A. fabae* was significantly less likely to initiate phloem sap ingestion (E2 activity, right panel of **B**) on virus-infected plants than on mock-inoculated plants. The infection status of plants made no significant difference to the already very low probability with which *M. persicae* re-initiates phloem sap ingestion (E2 activity, right panel of **B**). The survivorship functions, S(t), were calculated as Kaplan-Meier estimates and Kaplan-Meier p-values are shown (see **Supplementary Information Excel File 3** for pairwise curve comparisons).

Patterns of salivation into the phloem and ingestion of phloem sap (indicated by, respectively, waveforms E1 and E2) were further analyzed by survival analysis. For this analysis, the event of interest was the entry of *M. persicae* or *A. fabae* mouthparts into the phloem of mock-inoculated or virus-infected bean plants. A comparison of the two aphid species on uninfected plants, in terms of species differences in phloem salivation (E1) and phloem ingestion (E2) activity, showed that while aphids of both species were likely to penetrate and salivate in the phloem, *M. persicae* seemed innately reluctant to feed from common bean phloem compared to *A. fabae* and that this difference in species behavior was statistically significant (*p =* 0.00037) (**Figure 5A**).

For *A. fabae*, entry into the E2 phloem phase was significantly delayed on virus-infected plants (Kaplan-Meier: *p =* 0.017), and pairwise comparisons of *A. fabae* on CMV-infected compared with mock-inoculated plants were highly significant [Pairwise Peto-Peto: *p =* 0.0099 (**Figure 5B**, **Supporting Information: Excel File 3**)]. By comparison, there were no appreciable differences in the times taken by *M. persicae* to initiate phloem salivation or to initiate phloem feeding on mock-inoculated or virus-infected plants (**Figure 5C**, **Supporting Information Excel File 3**). Fewer *A. fabae* transitioned to sustained phloem-feeding on virus-infected plants than on mock-inoculated plants (**Figure 5B**). Of 15 aphids recorded in each treatment, 12 aphids on uninfected plants transitioned to sustained phloem feeding. In comparison, only 7, 8, and 5 aphids on BCMNV-, BCMV-, and CMV-infected plants, respectively, transitioned to sustained phloem feeding over the 8-h recording period (**Supporting Information: File 3 Sheet 2**). For *M. persicae,* only two aphids out of 15 in each treatment transitioned to sustained phloem feeding (**Figure 5C**, **Supporting Information: File 3 Sheet 2**).

### Aphids Experience Increased Mechanical Stylet Difficulties on Virus-Infected Plants

**Figure 6:**
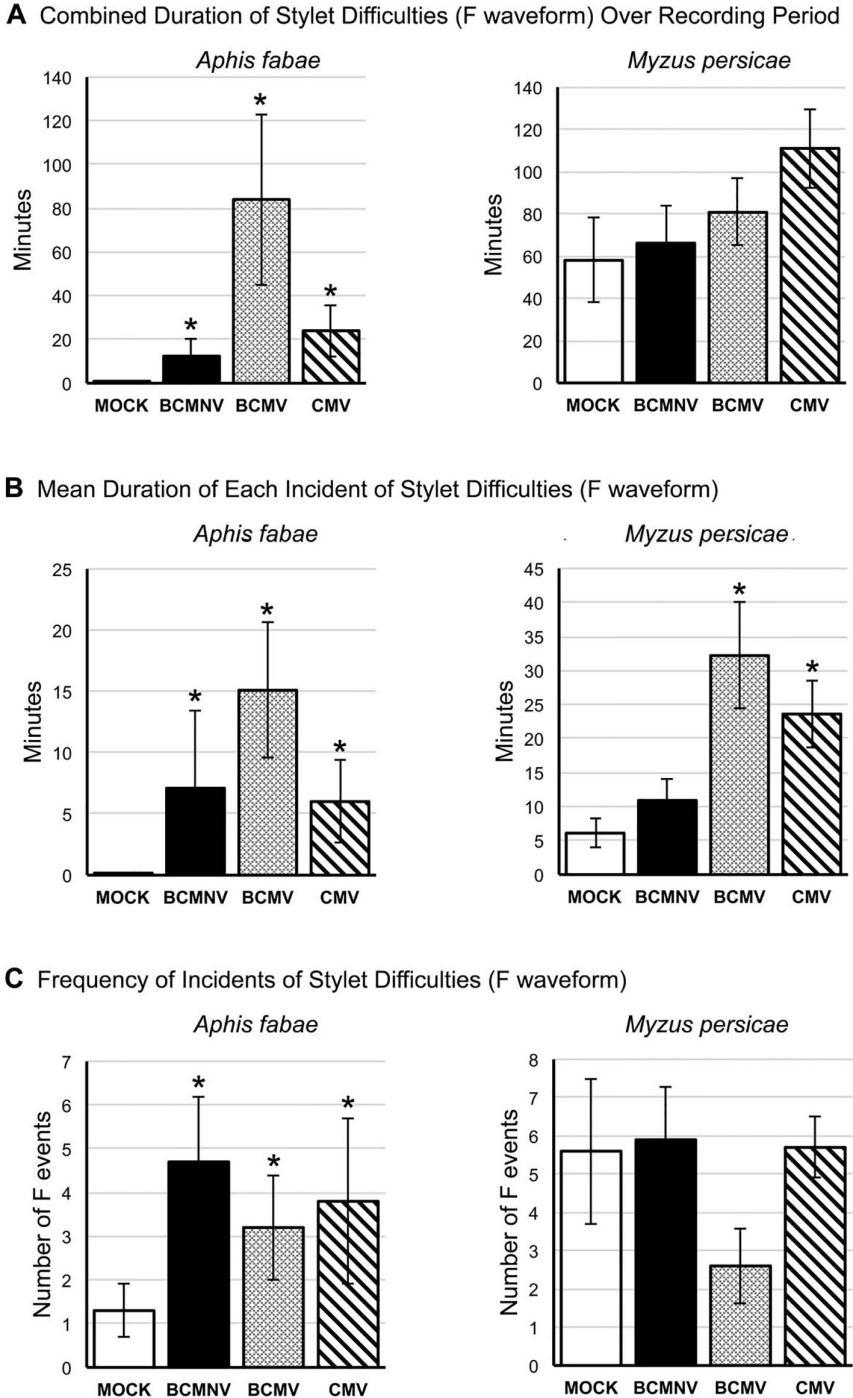
Mechanical stylet difficulty during aphid feeding is more frequently encountered by aphids placed on virus-infected plants. EPG data was analyzed to reveal: **(A)** the overall duration of time over the recording period in which aphids (the legume specialist, *Aphis fabae*, or the generalist, *Myzus persicae*) encountered stylet mechanical feeding difficulties (Waveform F: **Table 1**); **(B)** the mean duration of instances of feeding difficulty; and **(C)** the frequency of these feeding difficulty incidents over the recording period on mock-inoculated plants or plants infected with BCMNV, BCMV, or CMV. In contrast to their feeding on mock-inoculated plants *A. fabae* encountered more feeding difficulties on virus-infected plants (**A**, left panel) that were, in addition, more protracted **(B)** and more frequent **(C)**. The non-specialist aphid *M. persicae* experienced feeding difficulties even on mock-inoculated common bean plants and there was no overall increase in time spent experiencing feeding difficulties or in the frequency of these events on virus-infected plants as was the case for *A. fabae* **(A, C)**. However, *M. persicae* experienced more prolonged incidents of feeding difficulty on plants infected with BCMV and CMV (**B**, right panel). Asterisks denote significant differences in stylet difficulty between insects on virus-infected and mock-inoculated plants (Dunnett test, p < 0.05: see **Supplementary Table 1** for analyses). Error bars represent the standard error of the mean.

The EPG F waveform registers mechanical stylet activities occurring extracellularly and can indicate resistance to feeding. For *A. fabae,* more aphids showed mechanical stylet probing difficulties while feeding on virus-infected plants during the first 6 h of the recording period (**Supplementary Figure 3**). The total and mean duration of F waveform events were significantly increased in all virus treatments when compared to recordings done on mock-inoculated plants (**Figure 6**, **Supplementary Table 1**). There was a trend towards increased duration of feeding difficulties for *M. persicae* on virus-infected plants over the course of recording though this was not statistically significant. However, analysis of the mean duration of waveform F incidents indicated that feeding difficulties were significantly increased for *M. persicae* on plants infected with BCMV and CMV (**Figure 6**).

## Discussion

Aphids of both species tested, *A. fabae* and *M. persicae*, were quicker to commence probing on virus-infected bean plants, suggesting that virus-induced changes in the host influence the feeding behavior of aphids in a way likely to encourage virus acquisition. The increased speed with which both the non-specialist (*M. persicae*) and legume specialist (*A. fabae*) aphids commenced probing on virus-infected plants suggests that all three viruses induce changes at the leaf surface, which could include volatile chemical signals, that encourage probing of epidermal cells by aphids regardless of their specialisation. For *A. fabae*, both the overall number of probes and the number of short probes lasting less than 3 min increased on virus-infected plants, a pattern which will favor enhanced virus acquisition for all three of these non-persistently transmitted viruses (Powell, 2005; Krenz et al., 2015). Experiments with CMV-infected cucumber have also shown an increase in the number of probes by *A. gossypii* aphids especially in the initial stages of their EPG recording (Carmo-Sousa et al., 2014). These increases in probing activity would also promote transmission of these viruses. The “potential drop” (Pd, **Table 1**) activity in the short probes, occurring when the aphid penetrates the epidermal cells are important in the efficient transmission of potyviruses (Powell et al., 1995; Moreno et al., 2012).

EPG showed that on bean plants infected with BCMV, BCMNV, or CMV, *A. fabae* phloem feeding was inhibited and fewer aphids transitioned to sustained bouts of phloem feeding than did *A. fabae* placed on uninfected plants. These findings are consistent with those seen for *A. gossypii* on CMV-infected cucumber where both phloem salivation (E1) and feeding (E2) was reduced on infected plants (Carmo-Sousa et al., 2014). Usually, aphids can overcome impediments to phloem feeding, such as callose deposition, by salivating into the plant phloem during the E1 phase (Will et al., 2007). This appears hampered, especially on virus-infected plants, and the resulting reduced phloem feeding is unfavorable to long term colonization. The ensuing dispersal to more favorable hosts would promote local vectoring of non-persistently transmitted viruses (Donnelly et al., 2019). On plants infected with any of the three viruses, *A. fabae* experienced mechanical stylet difficulties, whereas these difficulties did not occur on mock-inoculated plants. The biochemical mechanisms underpinning this are yet to be investigated but, presumably, virus infection induces the production of feeding deterrent compounds or barriers in or around the phloem such as callose deposition. However, the discouragement of phloem feeding caused by the mechanical difficulties is also likely to enhance virus transmission.

Examination of *M. persicae* feeding behavior on virus-infected and on mock-inoculated plants suggested that these aphids were less likely than *A. fabae* to select common bean as a long-term host regardless of whether the plant was virus-infected or not. Based on the examination of phloem feeding activities (E1 and E2), only two of 15 aphids on either the mock-inoculated plants or the virus-infected plants transitioned into long-term phloem feeding. Analysis of the E2 waveform, which is indicative of phloem feeding and is a measure of plant acceptance, and of the incidence of stylet difficulties on mock-inoculated plants, led us to conclude that *M. persicae*, though a generalist aphid, found common bean unsuitable as a host upon which it would settle and feed. The results suggest that *M. persicae* would be unlikely to colonise common bean if other host plants are available. In previous EPG studies conducted by this group on tobacco, which is amenable to colonization by *M. persicae*, higher proportions of aphids showed sustained phloem ingestion on CMV-infected plants than on plants inoculated with a CMV mutant unable to express of the 2b protein (CMVΔ2b) (Ziebell et al., 2011). A subsequent study on Arabidopsis and *M. persicae* showed that infection with a severe strain of CMV (“Fast New York”; Fny-CMV) caused reduction of phloem feeding (Westwood et al., 2013). These studies showed that certain viral gene products might elicit aphid resistance and differential responses depending on the host plant. Also, within an aphid species where there are different races, plant host factors play a role in determining colonization (Schwarzkopf et al., 2013). Although common bean is unlikely to be colonized by *M. persicae*, as shown from our results, the overall feeding behavior is consistent with the ability to vector non-persistently transmitted viruses of bean. Our own observations from a previous study in Kenya found that under field conditions, the predominant aphid species present in bean plots was *A. fabae*, and *M. persicae* was not present (Wamonje et al., 2017). However, in other locations, *Myzus persicae* aphids have been found on field-grown bean plants infected with CMV and experimentally these aphids have been determined to potential effective vectors for the virus (Gildow et al., 2008).

In conclusion, virus-induced effects on aphid phloem feeding behavior were most evident for the bean specialist aphid (*A. fabae*) than for the generalist, *M. persicae*. Disruption of sustained phloem sap ingestion, especially on CMV-infected plants, coupled with enhanced mechanical stylet difficulty when probing, are likely to favor aphid migration from infected to more suitable (uninfected) plant hosts. These virus-induced effects are likely to contribute to the spread of BCMV, BCMNV, and CMV between common bean plants. Breeding for virus-resistant bean varieties has been the main strategy in controlling the spread of BCMV and BCMNV. This approach is threatened by the emergence of recombinant strains able to break current recessive gene stacking techniques (Feng et al., 2014; Feng et al., 2015). There are no known resistance genes effective for bean-infecting strains of CMV. Bean-infecting CMV isolates have been associated with major crop losses in the US (Thompson et al., 2015). In Kenya, metagenomic techniques identified reassortant CMV strains that were of Asian origin and had presumably arrived relatively recently *via* trade routes (Mutuku et al., 2018). Therefore, control strategies that target the vectoring of CMV are important, as well as measures to prevent the accidental introduction of novel strains into new localities. Our results may be useful in supporting these strategies. We further suggest that improve dagronomic practices that reduce sources of the virus in the field, and methods for production or that inhibit aphid-mediated transmission, as well as methods to produce virus-free bean seed, need to be developed.

## Data Availability Statement

All datasets generated for this study are included in the article/**Supplementary Material**.

## Author Contributions

JPC, FW, JP, TB, and CG planned and designed the research. FW, TT, AM, AP, and JC performed experiments. FW, RD, CG, TB, CW, JM, JP, AM, and JPC analyzed the data. FW, AM, and JPC wrote the manuscript with contributions from all authors.

## Conflict of Interest

The authors declare that the research was conducted in the absence of any commercial or financial relationships that could be construed as a potential conflict of interest.

## Supplementary Material

Table S1 Raw EPG data for Aphis fabae feeding behavior for **Figure 2**.

Table S2 Raw EPG data for Myzus persicae feeding behavior for **Figure 2**.

Table S3 Survivorship analysis of aphid phloem feeding behavior for **Figure 5**.
