# Supplementary material for: Different Plant Viruses Induce Changes in Feeding Behavior of Specialist and Generalist Aphids on Common Bean That Are Likely to Enhance Virus Transmission
Source: Front Plant Sci. 2020 Jan 31;10:1811. doi: 10.3389/fpls.2019.01811 (PMC7005137; doi:10.3389/fpls.2019.01811)
Supplement: Supplementary file 4 [file DataSheet_1.pdf]

Supplementary Data Sheet 1

Different plant viruses induce changes in feeding behaviour of specialist and generalist aphids on common bean that are likely to enhance virus transmission

Francis O. Wamonje[1, ♮], Ruairí Donnelly[1], Trisna D. Tungadi[1], Alex M. Murphy[1], Adrienne E. Pate[1], Christine Woodcock[2], John Caulfield[2], J. Musembi Mutuku[1,3], Toby J. A. Bruce[2*], Christopher A. Gilligan[1], John A. Pickett[2§] and John P. Carr[1#].

1. Department of Plant Sciences, University of Cambridge, Cambridge CB2 3EA, UK

2. Rothamsted Research, Harpenden, Hertfordshire AL5 2JQ, UK

3.Biosciences eastern and central Africa, International Livestock Research Institute, P.O. Box 30709-00100, Nairobi, Kenya.

[#] Corresponding author: jpc1005@hermes.cam.ac.uk

[♮]Present address: International Centre of Insect Physiology and Ecology, 30772-00100 Nairobi, Kenya

*Present address: School of Life Sciences, Keele University, Staffordshire ST5 5BG, UK

§ Present address: School of Chemistry, Cardiff University, Cardiff, CF10 3AT, UK

Supplementary Table 1. Analysis of mechanical stylet difficulties and xylem ingestion (mean ± SEM) of *Aphis fabae* and *Myzus persicae* on virus-infected and mock-inoculated bean plants over an 8-hour EPG recording.

Aphid	EPG Parameters (waveform[#])		Unit	Mock[§]	BCMNV	BCMV	CMV
Aphis fabae	**Mechanical stylet difficulty (F)**	Cumulative Total Duration of F	Min	0.4 ±0.1	12.4 ±7.7* [*p* = $3.11.10^{-5}$]	84 ±39* [*p*=$7.92.10^{-11}$]	24 ±12* [*p*= 1.6610^{-6}]
		Frequency of F	Number	1.3 ±0.6	4.7 ±1.5	3.2 ±1.2	3.8 ±1.9
		Mean length of incidents of F waveform	Min	0.1 ±0.01	7.1 ±6.3* [*p*= $2.46.10^{-5}$]	15.1 ±5.5* [*p*=$8.09.10^{-7}$]	6 ±3.4* [*p*= $4.16.10^{-5}$]
	**Xylem ingestion (G)**	Cumulative Total Duration of G	Min	50 ±10.5	69 ±14.6	53 ±10	41 ±13.4
		Frequency of G	Number	0.6 ±0.2	1 ±0.2	0.7 ±0.2	0.4 ±0.2
		Mean length of G	Min	48 ±11	55 ±13	45 ±10	40 ±14
Myzus persicae	**Mechanical stylet difficulty (F)**	Cumulative Total Duration of F	Min	58 ±20	66.1 ±17.7	80.7 ±15.8	111 ±18.7
		Frequency of F	Number	5.6 ±1.9	5.9 ±1.4	2.6±1	5.7 ±0.8
		Mean length of incidents of F waveform	Min	6.1 ±2.1	10.9 ±3.1	32.2±7.8* [*p*= 0.00019]	23.6±4.9* [*p* = 0.0011]
	**Xylem ingestion (G)**	Cumulative Total Duration of G	Min	60.5 ±11.1	86 ±13.7	71.8 ±13.6	52.1 ±10.9
		Frequency of G	Number	1.9 ±0.4	3 ±0.6	1.9 ±0.3	1.4 ±0.4
		Mean length of incidents of G waveform	Min	34.6 ±6.3	31.8 ±5.8	37.2±5.2	26.4±4

Asterisk (*) indicates Bonferroni corrected *p* values where significant.

[#] For full descriptions of EPG waveforms see Table 1 and Supplementary Figure 1.

[§] Mock = mock-inoculated *v.* plants infected with bean common mosaic necrosis virus (BCMNV), bean common mosaic virus (BCMV), or cucumber mosaic virus (CMV).

Supplementary Figures

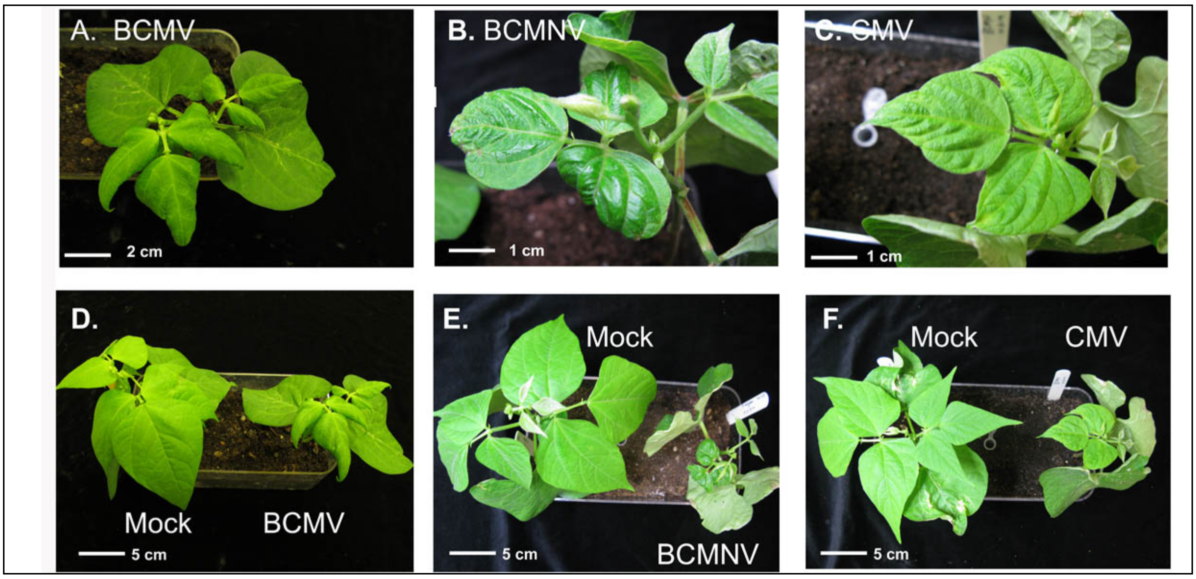

Supplementary Figure 1. Viral disease symptoms on common bean. The upper panels (A - C) are photographs of typical foliar symptoms on common bean (*Phaseolus vulgaris*) plants infected with bean common mosaic virus (BCMV), bean common mosaic necrosis virus (BCMNV) and cucumber mosaic virus (CMV). Lower panels (D - F) show the virus-infected plants and mock-inoculated (Mock) plants for comparison. All the viruses caused clearly observable disease symptoms on common bean. Plants were photographed at 10 days post-inoculation or mock-inoculation.

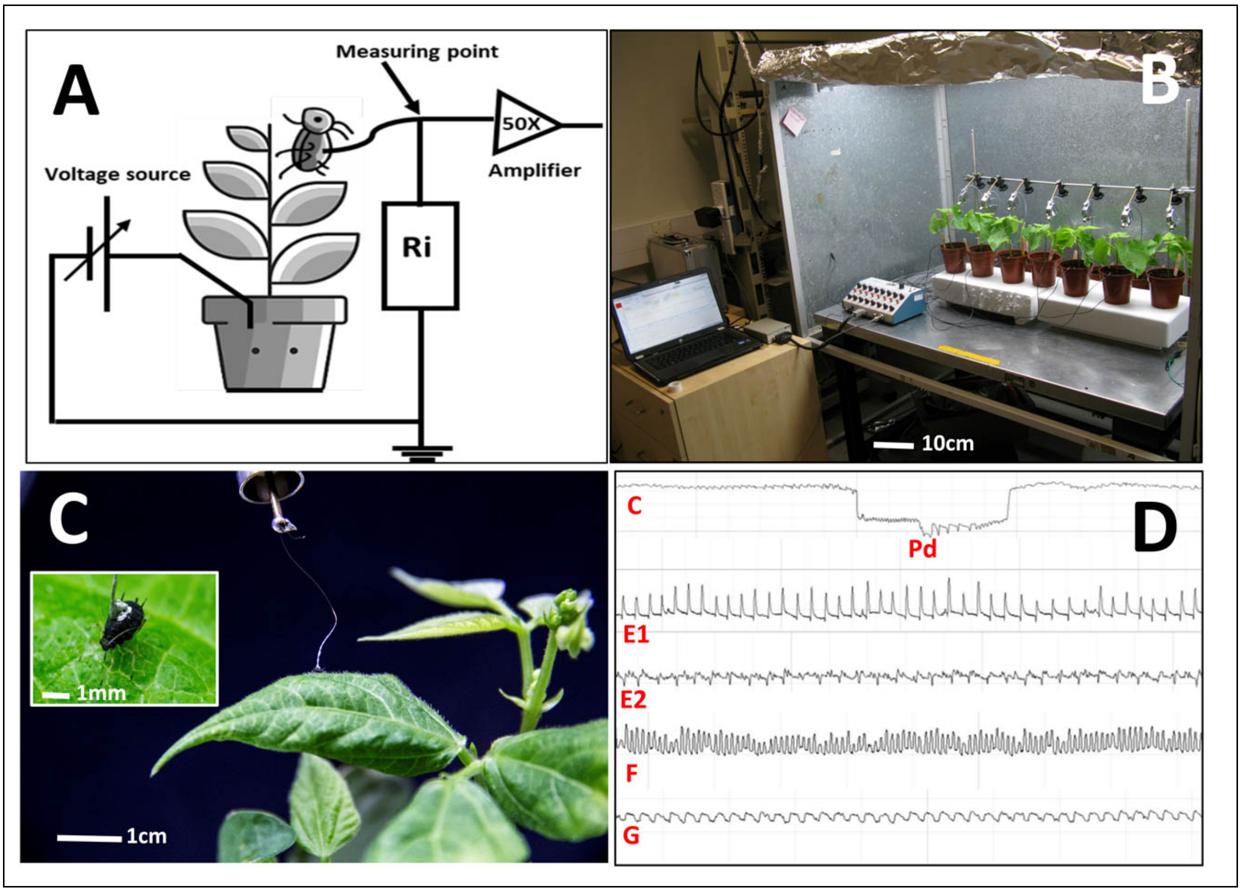

Supplementary Figure 2. Monitoring of aphid feeding behaviour by the electrical penetration graph (EPG) method. Panel (**A**) illustrates the EPG set up (not to scale) (adapted from Tjallingii, 2006). Panel (**B**) shows the EPG apparatus within a Faraday cage with the computer placed outside to minimise electrical interference. (**C**) The panel shows an aphid connected to the terminal and placed on a bean leaf. The inset shows a close-up of an aphid on the bean leaf surface and the gold wire connected by silver-containing glue to the dorsum. Panel (**D**) shows the different waveforms (marked as C, Pd, E1, E2, F and G) elicited by aphids.

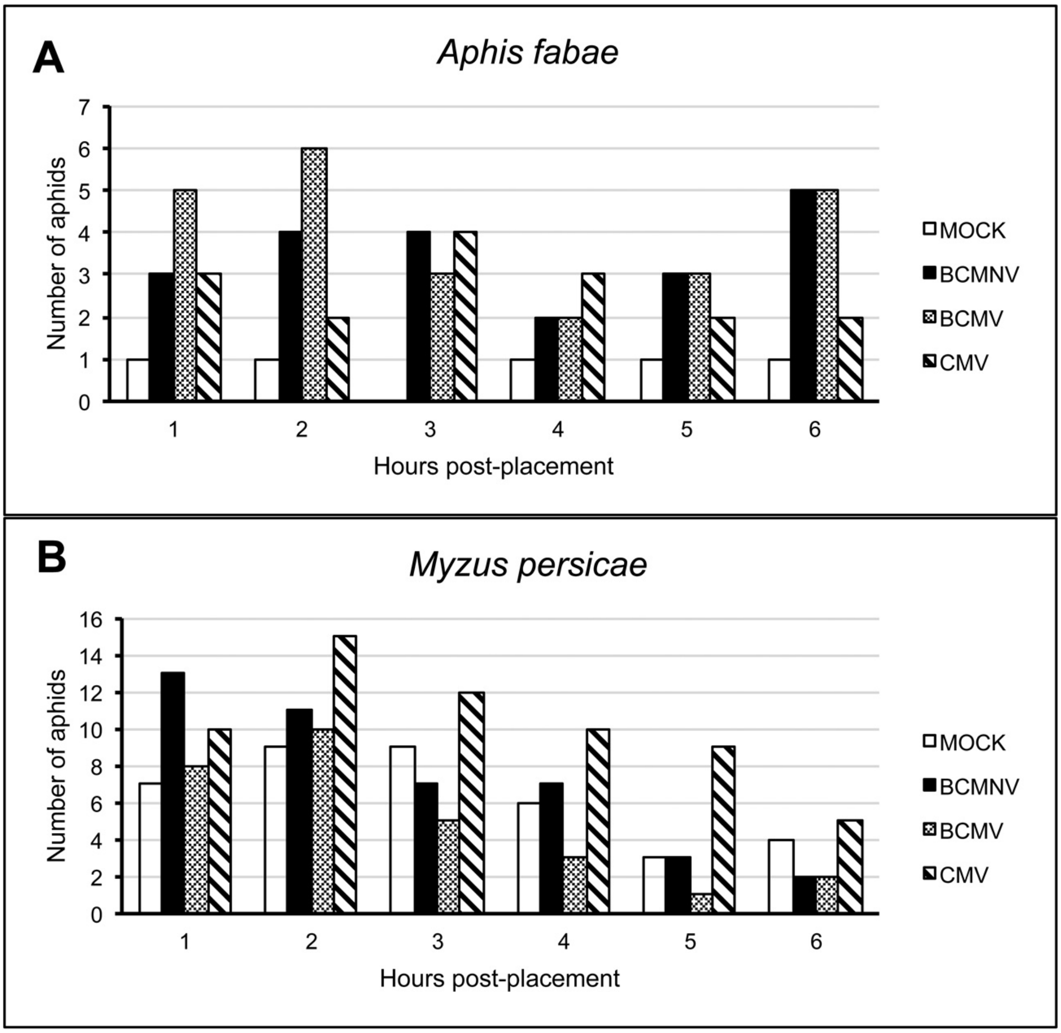

Supplementary Figure 3. Mechanical stylet difficulty (Waveform F) in aphid feeding occurs more frequently on virus-infected bean plants. EPG showed that more aphids of the species *A. fabae* experienced mechanical stylet difficulties (Waveform F: Table 1 and Fig. S1) when feeding on virus-infected plants over the first 6 h of recording following placement of aphids on plants (A). For *M. persicae* the number of aphids experiencing mechanical stylet difficulties was higher when feeding on plants infected with any of the three viruses only during the first hour of EPG recording (B). However, consistently over the 6-hour recording period, more aphids exhibited mechanical stylet difficulty on CMV-infected plants than on mock-inoculated plants. Mock = mock-inoculated plants; CMV = cucumber mosaic virus-infected plants; BCMV = bean common mosaic virus-infected plants; BCMNV = bean common mosaic necrosis virus-infected plants.
